# Single- and double-walled carbon nanotubes enhance atherosclerogenesis by promoting monocyte adhesion to endothelial cells and endothelial progenitor cell dysfunction

**DOI:** 10.1186/s12989-016-0166-0

**Published:** 2016-10-13

**Authors:** Yuka Suzuki, Saeko Tada-Oikawa, Yasuhiko Hayashi, Kiyora Izuoka, Misa Kataoka, Shunsuke Ichikawa, Wenting Wu, Cai Zong, Gaku Ichihara, Sahoko Ichihara

**Affiliations:** 1 Graduate School of Regional Innovation Studies, Mie University, 1577 Kurimamachiya-cho, Tsu, 514-8507 Japan; 2 Graduate School of Natural Science and Technology, Okayama University, Okayama, Japan; 3 Department of Occupational and Environmental Health, Nagoya Univeristy Graduate School of Medicine, Nagoya, Japan; 4 Department of Occupational and Environmental Health, Tokyo Univeristy of Science, Noda, Japan

**Keywords:** Carbon nanotubes, Atherosclerosis, Endothelial cells, Adhesion, Endothelial progenitor cells

## Abstract

**Background:**

The use of carbon nanotubes has increased lately. However, the cardiovascular effect of exposure to carbon nanotubes remains elusive. The present study investigated the effects of pulmonary exposure to single-walled carbon nanotubes (SWCNTs) and double-walled carbon nanotubes (DWCNTs) on atherosclerogenesis using normal human aortic endothelial cells (HAECs) and apolipoprotein E-deficient (ApoE^−/−^) mice, a model of human atherosclerosis.

**Methods:**

HAECs were cultured and exposed to SWCNTs or DWCNTs for 16 h. ApoE^−/−^ mice were exposed to SWCNTs or DWCNTs (10 or 40 μg/mouse) once every other week for 10 weeks by pharyngeal aspiration.

**Results:**

Exposure to CNTs increased the expression level of adhesion molecule (ICAM-1) and enhanced THP-1 monocyte adhesion to HAECs. ApoE^−/−^ mice exposed to CNTs showed increased plaque area in the aorta by oil red O staining and up-regulation of ICAM-1 expression in the aorta, compared with vehicle-treated ApoE^−/−^ mice. Endothelial progenitor cells (EPCs) are mobilized from the bone marrow into the circulation and subsequently migrate to the site of endothelial damage and repair. Exposure of ApoE^−/−^ mice to high-dose SWCNTs or DWCNTs reduced the colony-forming units of EPCs in the bone marrow and diminished their migration function.

**Conclusion:**

The results suggested that SWCNTs and DWCNTs enhanced atherosclerogenesis by promoting monocyte adhesion to endothelial cells and inducing EPC dysfunction.

**Electronic supplementary material:**

The online version of this article (doi:10.1186/s12989-016-0166-0) contains supplementary material, which is available to authorized users.

## Background

Although humans have been exposed to airborne nano-sized particles throughout their evolutionary stages, such exposure has increased dramatically over the last century due to anthropogenic sources. Engineered nanomaterials and nanotechnologies are expected to have an impact on society and economy. There are also concerns that these materials may pose environmental and health risks due to their unusual chemical and physical properties [1, 2]. Thus, information about the safety and potential hazards of nanomaterials is urgently needed, because various engineered nanomaterials had already been incorporated into various industrial processes and products [3].

Evidence based on epidemiological and toxicological studies suggests that high concentrations of particles measuring <2.5 μm in diameter (PM2.5) are associated with increased risk of pulmonary complications, cardiovascular events, and death from cardiovascular diseases [4–7]. Ambient particulate pollutants in the ultrafine range have been also shown to enhance the early development of atherosclerosis [8]. It has been demonstrated that certain nanomaterials can generate reactive oxygen species (ROS), resulting in induction of oxidative stress and inflammation [9, 10]. We have reported that zinc oxide (ZnO) nanoparticles can potentially enhance the migration and adhesion of THP-1 monocytes to human umbilical vein endothelial cells (HUVECs) and uptake of modified LDL by THP-1 macrophages [11], suggesting that certain nanoparticles can advance atherosclerogenesis.

Engineered carbon nanomaterials have many properties, such as large surface area, high electrical conductivity, and excellent strength. Carbon nanomaterials are used in many applications, such as electronic components and monitors, drug delivery, and hydrogen storage [12]. Carbon nanotubes are categorized as a single-layer; single-walled carbon nanotubes (SWCNTs) and multi-walled carbon nanotubes (MWCNTs). The double-layer; double-walled carbon nanotubes (DWCNTs) are a specific subset of MWCNTs. DWCNTs are suitable for use as field-effect transistors and would be used for photoconversion and electrical energy storage that require high technology [13]. Recent studies demonstrated that exposure to SWCNTs is associated with increased ROS production in cultured endothelial cells [14] and that exposure to MWCNTs have cytotoxic and genotoxic effects on HUVECs probably through oxidative damage [15]. However, the effects and the mechanisms of CNTs on the cardiovascular system remain undefined.

Previous studies demonstrated the translocation and accumulation of several types of nanoparticles in the bone marrow following their administration in laboratory animals [16, 17]. Since endothelial progenitor cells (EPCs) play an important role in facilitating vascular repair and tissue regeneration [18], we hypothesized that nanomaterials have certain effects on the function of EPCs. The present study investigated the effects of CNTs on the adhesion of monocytes, which is an essential process in atherosclerogenesis, using an in vitro set-up of normal human aortic endothelial cells (HAECs) and human monocytic leukemia cells (THP-1). We also examined the effects of exposure to CNTs on the progression of atherosclerosis and analyzed their ex vivo role on the function of isolated EPCs of bone marrow origin in apolipoprotein E deficient (ApoE^−/−^) mice, a widely used model of human atherosclerosis.

## Results

### Characterization of suspensions of CNTs

**Fig. 1:**
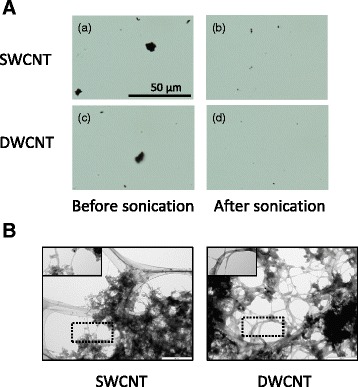
Optical microscope and TEM micrographs of CNT suspensions at 1.0 mg/ml concentration. **a** Optical microscope before and after dispersion by a cup-type sonicator at 100 W, 80 % pulse mode, for 10 min twice. **b** Transmission electron microscope (TEM) micrographs of SWCNTs and DWCNTs dispersed in the dispersion medium. The areas, which include well-dispersed CNTs, were highlighted within the dotted lines. SWCNT: single-walled carbon nanotube, DWCNTs: double-walled carbon nanotube

The intensity-weighted hydrodynamic average diameter of dispersed CNTs in the dispersion medium was measured by dynamic light scattering (DLS) technology. The mean hydrodynamic diameter and polydispersity index (PdI) were significantly different between dispersed SWCNTs and DWCNTs (Table 1). The Brunauer–Emmett–Teller (BET) surface area of SWCNTs was significantly larger than that of DWCNTs (Table 1). Highly agglomerated masses of CNTs were dispersed into small-size clusters and CNTs bundles were separated homogeneously by sonication (Fig. 1a). Transmission electron microscopy confirmed the presence of individual and bundled nanotubes in both sonicated CNTs (Fig. 1b).

**Table 1:** Physical characterization of CNTs

Materials	Hydrodynamic size (nm)	PdI	BET surface area (m^2^/g)
SWCNTs	134.5 ± 1.886	0.270 ± 0.010	646.0 ± 4.688
DWCNTs	128.6 ± 0.337*	0.218 ± 0.010*	527.5 ± 1.613*

Values are mean ± SD of 3–4 independent experiments

*SWCNTs* single-walled carbon nanotubes, *DWCNTs* double-walled carbon nanotubes, *PdI* polydispersity index

*p < 0.05 vs. SWCNTs

### Effects of CNTs on cell viability

**Fig. 2:**
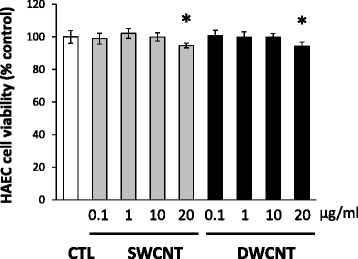
Effects of SWCNTs and DWCNTs on cell viability. Cell viability was measured by cell viability assay kit. HAECs were exposed to CNTs at concentrations ranging from 0.1 to 20 μg/ml for 16 h. Data are mean ± SD. (*n* = 8, *p < 0.05 vs. control; CTL) HAEC: normal human aortic endothelial cell, SWCNT: single-walled carbon nanotube, DWCNT: double-walled carbon nanotube

HAECs were exposed to SWCNTs or DWCNTs at a concentration ranging from 0.1 to 20 μg/ml for 16 h. The cell viability assay showed that incubation of HAECs in the presence of SWCNTs or DWCNTs at 20 μg/ml reduced cell viability (Fig. 2). When cell viability was determined after incubation at the final concentrations of CNTs ranged from 2.5 to 50 μg/ml for 16 h as a preliminary study, exposure to 25 and more μg/ml of SWCNTs or DWCNTs reduced cell viability in a dose-dependent manner (Additional file 1: Figure S1).

### Effects of CNTs on monocyte adhesion

**Fig. 3:**
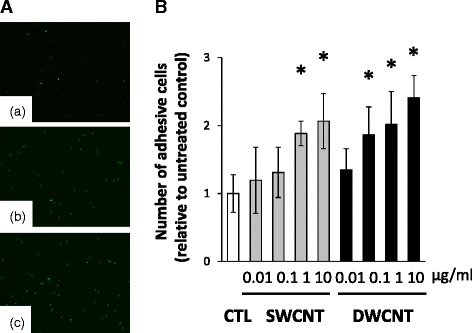
Adhesion assay of THP-1 monocytes to HAECs. **a** Representative images of THP-1 monocyte adhesion to HAECs. Panel (*a*) represents image under control conditions. (*b*) and (*c*) represent the adhesion of THP-1 cells to HAECs after exposure to 10 μg/ml of SWCNTs or DWCNTs for 16 h. **b** Relative number of THP-1 monocytes adherent to HAECs after exposure to SWCNTs or DWCNTs (0.01- 10 μg/ml) for 16 h. Data are mean ± SD. (*n* = 6,*p < 0.05 vs. control; CTL) SWCNT: single-walled carbon nanotube, DWCNT: double-walled carbon nanotube

We used the adhesion assay to test the effects of SWCNTs or DWCNTs on the adhesion of THP-1 monocytes to HAECs. The number of THP-1 cells that adhered to HAECs was significantly increased in the presence of 1, and 10 μg/ml of SWCNTs and 0.1, 1, and 10 μg/ml of DWCNTs in a dose-dependent manner (Fig. 3).

### Effect on CNTs on expression of chemokine and adhesion molecules

**Fig. 4:**
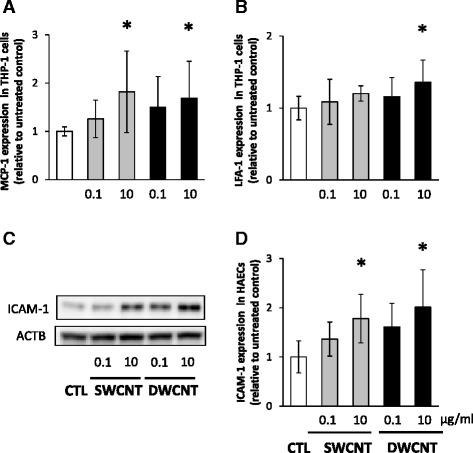
Expression of MCP-1 and LFA-1 in THP-1 monocytes and ICAM-1 in HAECs. **a** MCP-1 and **b** LFA-1 relative mRNA expression levels in THP-1 cells were determined 6 h after exposure to 0.1 or 10 μg/ml of SWCNTs or DWCNTs. Data are mean ± SD. (*n* = 8,*p < 0.05 vs. control; CTL) **c** Representative images of western blot analysis of ICAM-1 in HAECs exposed to 0.1 or 10 μg/ml of SWCNTs or DWCNTs. **d** Relative expression levels of ICAM-1 in HAECs exposed to SWCNTs or DWCNTs. Data are mean ± SD. (*n* = 6,*p < 0.05 vs. control; CTL) MCP-1: monocyte chemotactic protein-1, LFA-1: lymphocyte function-associated antigen 1, ICAM-1: intracellular adhesion molecule 1, SWCNT: single-walled carbon nanotube, DWCNT: double-walled carbon nanotube

Based on the above results, we examined the expression of chemokines and integrin to determine their roles in the adhesion of THP-1 monocytes. The expression of monocyte chemotactic protein-1 (MCP-1) was significantly higher in THP-1 cells exposed to 10 μg/ml of SWCNTs or DWCNTs than the control (Fig. 4a). Furthermore, exposure to 10 μg/ml of DWCNTs resulted in upregulation of lymphocyte function-associated antigen 1 (LFA-1) in THP-1 monocytes compared with the control (Fig. 4b). We also examined the expression of adhesion molecules in HAECs. Exposure to 10 μg/ml of SWCNTs or DWCNTs upregulated intracellular adhesion molecule 1 (ICAM-1) expression in HAECs compared with the control (Fig. 4c, d).

### Atherosclerogenesis in ApoE^−/−^ mice

**Fig. 5:**
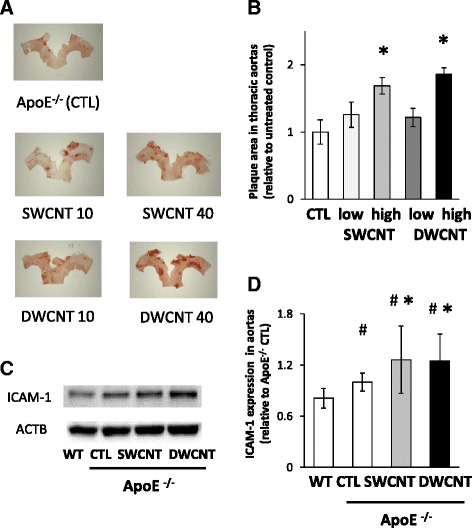
Plaque formation in the thoracic aorta of ApoE^−/−^ mice. **a** Representative images of thoracic aortas stained with oil red O solution of ApoE^−/−^ mice exposed to SWCNTs or DWCNTs. **b** Plaque area in the thoracic aortas. **c** Representative images of western blot analysis of ICAM-1 in ApoE^−/−^ mice repeatedly exposed to 40 μg of SWCNTs or DWCNTs. **d** Relative expression levels of ICAM-1 in the thoracic aorta of ApoE^−/−^ mice. Data are mean ± SD. (*n* = 5–7,*p < 0.05 vs. control of ApoE^−/−^ mice; CTL) ICAM-1: intracellular adhesion molecule 1, SWCNT: single-walled carbon nanotube, DWCNT: double-walled carbon nanotube

We assessed whether exposure to SWCNTs or DWCNTs by pharyngeal aspiration induces atherosclerogenesis in ApoE^−/−^ mice. ApoE^−/−^ mice were exposed chronically to the dispersion medium or CNTs (SWCNTs or DWCNTs at 10 or 40 μg/mouse) once every other week for 10 weeks. After 10 weeks, lung weight was significantly higher in ApoE^−/−^ mice exposed to 40 μg DWCNTs than the control (Table 2). There were no significant differences in body weight and weight of other organs (liver, kidney, spleen, and brain) between the groups. The percentage of the plaque area was determined. The extent of atherosclerosis was significantly larger in the thoracic aorta of ApoE^−/−^ mice exposed to the high dose of SWCNTs or DWCNTs than the control (Fig. 5a, b).

**Table 2:** Body and organ weights of mice exposed to CNTs

	C57BL/6 CTL	ApoE^−/−^				
		CTL	SWCNTs		DWCNTs	
			Low	High	Low	High
Body weight (g)	26.3 ± 2.5	29.2 ± 2.3	28.3 ± 1.4	30.4 ± 1.5	29.6 ± 2.5	27.9 ± 2.6
Lung weight (g)	0.16 ± 0.04	0.17 ± 0.02	0.18 ± 0.01	0.19 ± 0.02	0.20 ± 0.02	0.22 ± 0.01*
Liver weight (g)	1.26 ± 0.12	1.40 ± 0.22	1.35 ± 0.10	1.43 ± 0.10	1.37 ± 0.20	1.35 ± 0.20
Kidney weight (g)	0.16 ± 0.02	0.17 ± 0.02	0.16 ± 0.01	0.18 ± 0.02	0.17 ± 0.02	0.17 ± 0.02
Spleen weight (g)	0.07 ± 0.01	0.08 ± 0.01	0.07 ± 0.01	0.09 ± 0.01	0.08 ± 0.02	0.09 ± 0.03
Brain weight (g)	4.78 ± 0.10	4.74 ± 0.11	4.61 ± 0.07	4.67 ± 0.15	4.73 ± 0.10	4.72 ± 0.12

Values are mean ± SD of 5–7 independent experiments

*SWCNTs* single-walled carbon nanotubes, *DWCNTs* double-walled carbon nanotubes

*p < 0.05 vs. control of ApoE^−/−^ mice; CTL

Based on the in vitro results of CNTs-enhanced adhesion of monocytes to endothelial cells, we examined the expression of ICAM-1 in the aortas of ApoE^−/−^ mice. Exposure to the high dose of SWCNTs or DWCNTs significantly increased the expression level of ICAM-1 in ApoE^−/−^ mice than the control (Fig. 5c, d).

### Effects of CNTs on number and function of EPCs

**Fig. 6:**
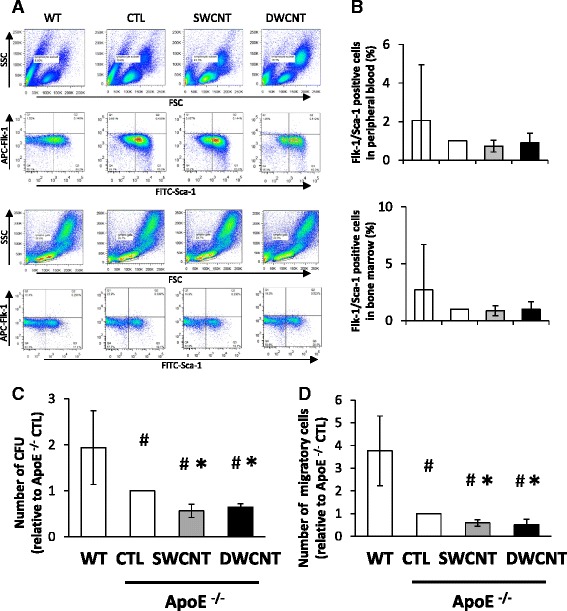
Number and function of EPCs in ApoE^−/−^ mice exposed to SWCNTs or DWCNTs. **a** Representative flow cytometry plots of side scatter (SSC) and forward scatter (FSC) or FITC-Scan-1^+^/APC-Flk-1^+^ cells isolated from peripheral blood or bone marrow, **b** Relative number of Flk-1^+^/Sca-1^+^ cells in peripheral blood or bone marrow, **c** number of colony-forming units, and **d** number of migratory cells after exposure to 40 μg of SWCNTs or DWCNTs. Data are mean ± SD. (*n* = 3,*p < 0.05 vs. control of ApoE^−/−^ mice; CTL) SWCNT: single-walled carbon nanotube, DWCNT: double-walled carbon nanotube

The atherosclerogenesis process is influenced by, in part, the number and functional status of EPCs [19]. Thus, we examined the effects of CNTs on the number and function of EPCs, as expressed by the colony-forming units (CFU) and migration ability. The numbers of Flk-1/Sca-1 positive cells in both the peripheral circulation and bone marrow were lower in ApoE^−/−^ control mice than wild-type control mice (Fig. 6a, b). Exposure to the high dose of SWCNTs and DWCNTs reduced the numbers of Flk-1/Sca-1 positive cells in peripheral blood and bone marrow of ApoE^−/−^ mice, but there were no significant differences between the groups (Fig. 6a, b). The number of CFU was significantly higher in wild-type control mice than ApoE^−/−^ control mice. Exposure to SWCNTs or DWCNTs significantly reduced the number of CFU in ApoE^−/−^ mice (Fig. 6c). Furthermore, the number of migratory EPCs in ApoE^−/−^ control mice was lower than in wild-type control mice, and exposure to SWCNTs or DWCNTs significantly decreased the number of migratory EPCs in ApoE^−/−^ mice (Fig. 6d).

## Discussion

The present study demonstrated that SWCNTs and DWCNTs enhanced the adhesion of THP-1 monocytes to HAECs through the up-regulation of expression of various adhesion molecules. We also demonstrated that exposure to SWCNTs and DWCNTs increased atherosclerotic plaque progression in ApoE^−/−^ mice. The results suggest that SWCNTs and DWCNTs enhance atherosclerogenesis by promoting the adhesion of monocytes to endothelial cells and inducing EPCs dysfunction.

The present results showed that 20 μg/ml of SWCNTs and DWCNTs each reduced HAECs cell viability. Previous studies reported that at 10 μg/ml or higher, MWCNTs reduced HUVECs cell survival [15]. At lower concentrations (50 or 150 μg/10^6^ cells, i.e., 1.5 or 4.5 μg/ml), SWCNTs and MWCNTs also induced significant LDH release which resulted in significant reduction in cytotoxicity [20]. Recent studies described carboxylated MWCNTs-induced decrease in HUVECs viability, associated with profound accumulation of autophagosomes [21]. Moreover, MWCNTs was also reported to increase ROS production in HUVECs at 2–16 μg/ml [22] and in human microvascular endothelial cells (HMVECs) at 2.5 μg/ml [23]. These results suggest that certain CNTs can induce damage of endothelial cells. The use of CNTs at concentrations less than 10 μg/ml in our experiments was based on the above findings of significantly reduced HAECs cell viability at 20 μg/ml.

SWCNTs and DWCNTs enhanced the adhesion of THP-1 monocytes to HAECs in the present study. Furthermore, both CNTs significantly increased intracellular concentrations of MCP-1 and LFA-1 in THP-1 monocytes. MCP-1 is known to play an important role in the early recruitment of monocytes to atherosclerotic lesions [24] and LFA-1 is the main integrin in leukocytes and an important molecule in firm adhesion and migration of leukocytes to the inflammatory sites [25]. LFA-1 plays pivotal roles as a signal transduction molecule by binding its ligand, namely, ICAM-1 [26]. In the present study, CNTs also significantly up-regulated ICAM-1 expression in HAECs. These results are consistent with those of previous studies that showed MWCNTs-induced increase in ICAM-1 expression in endothelial cells [22, 23]. ICAM-1 expression was also increased in the aorta tissue of ApoE^−/−^ mice exposed to SWCNTs and DWCTs. Our results indicate that CNTs increased intracellular concentration of LFA-1 in monocytes and ICAM-1 in endothelial cells and induced adhesion of monocytes to endothelial cells, which could be one of the mechanisms responsible for the accelerated atherosclerogenesis induced by CNTs.

Our results demonstrated that SWCNTs and DWCNTs increased the area stained with oil red in the thoracic aorta in our mouse model of atherosclerosis. MWCNTs have been shown to induce structural and functional changes in the endothelium of Sprague–Dawley rat model of atherosclerosis [27]. Other studies in ApoE^−/−^ mice on high-fat diet also showed that instillation of SWCNTs resulted in a slight increase in the plaque area in the aorta [28], and that ApoE^−/−^ mice exposed to MWCNTs showed accelerated plaque progression in the aorta tissue [22]. However, another study showed that these effects were minimal [29]. Our results showed a significant increase in the plaque areas in mice treated with SWCNTs or DWCNTs but there were no significant differences in the plaque areas between the two CNTs. In this regard, SWCNTs and MWCNTs are known to have direct effects on endothelial cells and that these effects are dose-dependent for both CNTs [20]. However, our study showed that lower concentrations of DWCNTs, relative to those of SWCNTs, induced significant increases in lung weight, THP-1 monocyte adhesion to HAECs and expression of adhesion molecules. Oxidative stress and inflammatory effects of MWCNTs were reported to be associated with its surface area (BET) and length [30]. In the present study, BET surface area of DWCNTs was significantly smaller than that of SWCNTs, while length of DWCNTs was significantly longer than that of SWCNTs. Therefore, it is difficult to determine which of surface area or length is critical to biological responses of CNTs in the present study. Further studies are needed to understand the cause(s) for the differences in the effects of SWCNTs and DWCNTs on cardiovascular system.

EPCs represent one subset of progenitor cells that originate in the bone marrow and are mobilized to the circulation after birth. These cells play an important role in facilitating vascular repair and tissue regeneration [31, 32]. Previous clinical studies demonstrated a clear association between reduced number or function of circulating EPCs and increased cardiovascular risk [33, 34]. Furthermore, high concentrations of PM2.5 were associated with high risk of cardiovascular events and death from cardiovascular diseases [35]. Moreover, exposure to PM2.5 induced reversible vascular injury by suppression of circulating EPC density in human [35]. Circulating EPCs were also decreased following exposure to high levels of PM2.5 in mice [36]. Thus, exposure to air pollutants seems to have the general property of reducing circulating EPCs, but a significant reduction in EPC numbers was not observed in the present study following exposure to CNTs.

Given that several types of nanoparticles can translocate and accumulate in the bone marrow [16, 17], they could be taken up by bone marrow-derived mononuclear cells, thus explaining their direct effects on such cells. Experiments in cultured cells showed that supermagnetic iron oxide nanoparticles impaired EPC migration and promoted EPC adhesion [37]. We also demonstrated recently that zinc oxide nano/micro particles suppressed vasculogenesis in human endothelial colony-forming cells [38]. Moreover, ex vivo functional assessments of cultured EPCs from the bone marrow of mice exposed to nickel nanoparticle showed reduced EPC tube formation and chemotaxis [39]. We have not examined whether CNTs were observed in the bone marrow or not in the present study. However, the present study demonstrated that SWCNTs and DWCNTs significantly reduced the number of CFU and decreased the migration of EPCs in response to vascular endothelial growth factor (VEGF) in ApoE^−/−^ mice. Moreover, Patlolla et al. [40] recently demonstrated the induction of oxidative stress mediated genotoxicity in bone marrow collected from the mice exposed to SWCNTs. Considered together, these results suggest that CNTs impair EPC functional activities. After vascular injury, EPCs are recruited from the bone marrow to peripheral blood by VEGF. Our findings suggest that CNTs enhanced atherosclerogenesis by, at least in part, reducing EPC function. However, the number of colony-forming units and migratory cells were measured in bone marrow isolated from the only three mice of each group. Further studies are needed to identify the effects of CNTs on function of EPCs.

Nano-sized particles have a possibility to cross the pulmonary epithelial barrier and enter the bloodstream [41, 42]. After inhalation, MWCNTs translocated into the bloodstream and then accumulated in body organs [43]. These results suggest that translocation to the peripheral circulation is a probable mechanism for the direct effect of these nanomaterials on the cardiovascular system. However, the present mice were exposed by pharyngeal aspiration to CNTs one a week for 10 weeks. It is possible that non-physiological phenomenon is induced by bolus exposure to CNTs. Inhalation studies are ideally required to conclude the present results.

Our results showed significant increases in the plaque area of mice exposed to SWCNTs or DWCNTs to the same extent. However, some parameters, such as lung weight, THP-1 monocyte adhesion to HAECs, and expression of adhesion molecules, were significantly increased by exposure to DWCNTs at the concentration lower than the concentration at which SWCNTs induced the same effects. This difference between SWCNTs and DWCNTs might be considered when establishing the exposure limit in occupational or environmental setting.

## Conclusions

The present study investigated the effects of CNTs on the adhesion of monocytes, an important process in atherosclerogenesis, using an in vitro set-up of HAECs and THP-1 cells. We also examined the effects of CNTs on atherosclerogenesis and analyzed their effects on the function of EPCs isolated from the bone marrow of ApoE^−/−^ mice, a model of human atherosclerosis. The results suggested that SWCNTs and DWCNTs enhanced atherosclerogenesis through the promotion of monocyte adhesion to endothelial cells and induction of EPCs dysfunction.

## Methods

### CNTs preparation and characterization

Single-walled carbon nanotubes (SWCNT; Nanocyl, Sambreville, Belgium) with an average diameter of 2 nm and length of several μm and double-walled carbon nanotubes (DWCNT, Nanocyl) with an average diameter of 3.5 nm and length of 1–10 μm were used in this study. CNTs were suspended in a dispersion medium and dispersed using sonicator (BRANSON Sonifier model 450, Danbury, CT; 80 % pulsed mode, 100 W, 10 min, twice), as described previously [44]. The dispersion medium comprised Ca^+2^- and Mg^+2^-free phosphate-buffered saline (PBS, pH 7.4), supplemented with 5.5 mM D-glucose and 0.6 mg/ml bovine serum albumin. The hydrodynamic sizes of the CNTs in the medium were measured four times after 1 h on standing using the DLS technology with a Zetasizer Nano-S (Malvern Instruments, Worcestershire, UK). The dispersion status was described by the intensity-weighted hydrodynamic average diameter (z-average) and PdI, which reflects the broadness of the size distribution (scale range from 0 to 1, with 0 being monodispersion and 1 being polydispersion) [45]. The BET surface area of CNTs was measured three times using the surface area analyzer with a BELSORP-mini II (Microtrac BEL, Osaka, Japan). CNTs suspension was viewed using an Olympus BXJ1 optical microscope (Olympus, Tokyo, Japan) equipped with a digital camera DP70, to capture images with the DP controller software (Olympus). Dispersed CNTs were visualized using a transmission electron microscope (TEM, JEM-1011; JEOL, Tokyo, Japan).

### Cell culture

HAECs from Lonza Group (Basel, Switzerland) were cultured in endothelial basal medium (EBM)-2 at 37 °C in 5 % CO_2_. Experiments were performed using the cells at passage 4 to 6. THP-1 cells from the American Type Culture Collection (ATCC, Rockville, ML) were cultured in RPMI 1640 medium (Life Technologies, Carlsbad, CA) containing 10 % FBS, penicillin (100 U/ml), streptomycin (100 μg/ml), and 50 μM 2-mercaptoethanol at 37 °C in 5 % CO_2_.

### Cell viability assay

HAECs were seeded at 1.0 × 10^4^ cells per well on 96-well plates overnight prior to the experiment. CNTs were dispersed in dispersion medium and the final concentrations of CNTs ranged from 0.1 to 20 μg/ml. Cell viability was determined after incubation of dispersed CNTs for 16 h as indicated by the CellTiter-Glo™ Luminescent Cell Viability Assay (Promega, Madison, WI). The effect of CNTs on cell proliferation was calculated as the percentage of inhibition of cell growth with respect to the controls.

### Cell adhesion assay

Adhesion of THP-1 cells to HAECs was assessed as described in detail previously [13]. Briefly, HAECs (1.0 × 10^4^ cells) were grown overnight in 96-well plates at 37 °C. The cells were exposed to different concentrations (0.01, 0.1, 1, or 10 μg/ml) of SWCNTs or DWCNTs for 16 h at 37 °C and prior to the adhesion assay, washed three times with Hank’s Balanced Salt Solution (HBSS) containing 0.1 % BSA. THP-1 cells were suspended at a density of 1.0 × 10^6^ cells/ml of 0.1 % BSA/HBSS and labeled with 1 μM of calcein-AM (BD Bioscience, Franklin Lakes, NJ) by 30 min incubation at 37 °C, followed by three washings with 0.1 % BSA/HBSS. The labeled THP-1 cells were then incubated with HAECs exposed to CNTs for 2 h at 37 °C. Nonadherent cells were removed carefully by three-time washings with 0.1 % BSA/HBSS. The adherence of calcein-labeled THP-1 cells was quantified by counting the number of endothelial monolayers using a fluorescent microscope (model FSX100, Olympus).

### Measurement of expression of chemokines and adhesion molecules

THP-1 cells were seeded at 2 × 10^5^ cells/well onto 6-well plates and exposed to 0.1 or 10 μg/ml of the dispersed CNTs for 6 h. The cells were collected by centrifugation at 1,000× rpm for 5 min at 4 °C. Total RNA from the cells was isolated by using ReliaPrep RNA cell miniprep system according to the protocol provided by the manufacturer (Promega). The concentration of total RNA was quantified by spectrophotometry (ND-1000; NanoDrop Technologies, Wilmington, DE). RNA was reverse transcribed to single-strand cDNA using SuperScript III First-Strand Synthesis System for RT-PCR (Life Technologies). The cDNA was subjected to quantitative PCR analysis with FastStart Universal Probe Master Mix (Roche, Basel, Switzerland) and primers for MCP-1 and LFA-1 using an ABI 7000 Real-Time PCR system (Life Technologies), as described previously [38]. The gene expression level was normalized to that of β-actin in the same cDNA.

For western blot analysis, HAECs were lysed in radioimmunoprecipitation assay (RIPA) lysis buffer containing protease inhibitors (Santa Cruz, Dallas, TX). The concentration of the extracted protein was measured in triplicate using the BCA Protein Assay Kit (Thermo Fisher Scientific, Waltham, MA). Protein samples were separated by 12 % SDS-PAGE and transferred onto polyvinylidene difluoride (PVDF) membranes (Immobilon-P, Millipore, Billerica, MA). The membranes were incubated with a rabbit monoclonal antibody to ICAM-1 (Abcam, Cambridge, MA) at a dilution of 1:500. Mouse anti-β-actin (ACTB) monoclonal antibody (Sigma-Aldrich, St Louis, MO) at dilution 1:5,000 was used as a loading control. Immunoreactive bands were visualized using ECL-select chemiluminescence reagent (GE Healthcare–Amersham, Buckinghamshire, UK) and the intensity of the bands was quantified by Quantity One v3.0 software (Bio-Rad Laboratories, Hercules CA). Protein expression levels were normalized relative to the level of β-actin protein in the same sample.

### Animal studies

B6.129P2-Apoe^tm1Unc^ (ApoE^−/−^) mice were obtained from Jackson Laboratory (Bar Harbor, ME). To evaluate the effects of CNTs on atherosclerogenesis, ApoE^−/−^ and wild-type mice (C57BL/6 J) (*n* = 5–7 in each group) were exposed by pharyngeal aspiration to 10 or 40 μg of SWCNTs or DWCNTs through multiple exposures (once a week) from 10 to 20 weeks of age (total amount administered was 100 or 400 μg). Exposure to cumulative dose of 128 μg MWCNTs induced atherosclerosis in one previous study, which examined plaque areas of ApoE^−/−^ exposed to MWCNTs, but exposure to cumulative dose of 640 μg MWCNTs did not in another study. Therefore, the present study set 100 or 400 μg as a cumulative dose. Body weight was measured once a week. All animal procedures were conducted in accordance with the guidelines for the care and use of laboratory animals approved by Mie University.

### Quantitative assessment of atherosclerosis

The thoracic aorta was harvested and fixed in PBS with 4 % paraformaldehyde and the adventitia was removed under a microscope, as described in detail previously [46]. Then, the aortic arch and the thoracic aorta were opened longitudinally, immersed for 1 min in 60 % isopropanol, and stained with oil red-O solution for 15 min at 37 °C. All images were captured with a microscope equipped with a camera (EZ4HD, Leica, Wetzlar, Germany) and analyzed using Image J Software. The edge of the aorta was traced using an automated feature and the extent of atherosclerosis was determined by selecting threshold ranges in the three basic colors of Image J Software. The total aortic surface area and the lesion area were then calculated. The extent of atherosclerosis was expressed as the percent of surface area of the aorta covered by lesions.

### Analysis of ICAM-1 production

The thoracic aorta was lysed in RIPA lysis buffer containing protease inhibitors. After measuring the concentration of the extracted proteins, the protein samples were separated by 12 % SDS-PAGE and transferred onto PVDF membranes. The membranes were incubated with rabbit monoclonal antibody to ICAM-1 (Abcam) at a dilution of 1:500. Mouse anti-β-actin (ACTB) monoclonal antibody (Chemicon International, Billerica, MA) at dilution 1:5,000 was used as the loading control. Immunoreactive bands were visualized using ECL-select chemiluminescence reagent, as described above.

### Measurement of number of EPCs in peripheral blood and bone marrow

Flow cytometry was applied for counting the number of EPCs (Sca-1^+^ and Flk-1^+^) in peripheral blood and bone marrow. Anticoagulated peripheral blood was obtained by decapitation. Bone marrow cells were obtained by flushing the tibias and femurs of mice with 2 % FBS/PBS. Next, 100 μl of peripheral blood or a volume of bone marrow suspension containing 1 × 10^6^ cells was immunolabeled with anti-Sca1-FITC (fluorescein isothiocyanate-conjugated stem cell antigen-1; BD Pharmingen, Franklin, NJ) and anti-Flk1-APC [allophycocyanin-conjugated fetal liver kinase-1 (VEGFR2, VEGF receptor 2), BD Pharmingen], as described previously [47]. Erythrocytes were lysed in FACS Lysing Solution (BD Pharmingen) and the remaining cells were analyzed by flow cytometry (FACS Canto II, BD Biosciences).

### Isolation of EPCs from bone marrow and colony-forming assay

Low-density bone marrow mononuclear cells were isolated by density centrifugation Histopaque-1083 (Sigma-Aldrich). For analysis of endothelial cell-colony forming units (EC-CFU), 2 × 10^6^ bone marrow-derived mononuclear cells were isolated and sub-cultured for 7 days in 20 % FBS/EBM-2 with supplements on human fibronectin pre-coated wells (including changing the culture medium every second day), as described previously [44]. After 7-day culture, the adherent cells were identified as EPCs by the uptake of 1,10-dioctadecyl-3,3,30,30-tetramethylindocarbocyanine-labeled acetylated LDL (DiLDL, 2.4 μg/mL; CellSystems, Troisdorf, Germany) and immunofluorescence staining of FITC-labeled Ulex europaeus agglutinin I (lectin, 10 μg/mL; Sigma-Aldrich). The number of colonies per well was counted manually.

### Migration assay

After counting the number of colonies, these cells were used for migration assay, as described previously in detail [48]. Briefly, the cells were first trypsinized, and then re-suspended in 20 % FBS/EBM-2. EPCs (2.0 × 10^3^ cells/well) were placed on the upper chamber of Cell Culture insert (8.0 mm pore size, 24-well plates; BD Falcon, Franklin, NJ, *n* = 3). EBM-2 and recombinant murine VEGF (50 ng/ml PeproTech, Rocky Hill, NJ) were harvested and used as the chemoattractant in the lower chamber of Cell Culture inserts and incubated for 24 h at 37 °C 5 % CO_2_. Cells that had actively migrated through the membrane were fixed by 4 % paraformaldehyde and transmigration was quantified using a fluorescent microscope; FSX100 (Olympus).

### Statistical analysis

All parameters were expressed as mean ± standard deviation (SD). Statistical analyses were performed using one-way analysis of variance (ANOVA) followed by Dunnett’s post hoc test. A *p* value less than 0.05 was considered statistically significant.

## Additional file

Additional file 1: Figure S1. Effects of SWCNTs and DWCNTs on cell viability. (PPTX 70 kb)

## Abbreviations

DWCNTs: Double-walled carbon nanotubes
EPCs: Endothelial progenitor cells
HAECs: Human aortic endothelial cells
ICAM-1: Intracellular adhesion molecule 1
LFA-1: Lymphocyte function-associated antigen-1
MCP-1: Monocyte chemotactic protein-1
SWCNTs: Single-walled carbon nanotubes

## References

[1] Colvin VL (2003). The potential environmental impact of engineered nanomaterials. Nat Biotechnol.

[2] Oberdörster G, Oberdörster E, Oberdörster J (2005). Nanotoxicology: an emerging discipline evolving from studies of ultrafine particles. Environ Health Perspect.

[3] Donaldson K, Stone V, Tran CL, Kreyling W, Borm PJ (2004). Nanotoxicology. Occup Environ Med.

[4] Mar TF, Norris GA, Koenig JQ, Larson TV (2000). Association between air pollution and mortality in Phoenix, 1995–1997. Environ Health Perspect.

[5] Miller KA, Siscovick DS, Sheppard L, Shepherd K, Sullivan JH, Anderson GL, Kaufman JD (2007). Long-term exposure to air pollution and incidence of cardiovascular events in women. N Engl J Med.

[6] Mills NL, Tornqvist H, Gonzalez MC, Vink E, Robinson SD, Söderberg S, Boon NA, Donaldson K, Sandström T, Blomberg A, Newby DE (2007). Ischemic and thrombotic effects of dilute diesel-exhaust inhalation in men with coronary heart disease. N Engl J Med.

[7] Peters A, Dockery DW, Muller JE, Mittleman MA (2001). Increased particle air pollution and the triggering of myocardial infarction. Circulation.

[8] Araujo JA, Barajas B, Kleinman M, Wang X, Bennett BJ, Gong KW, Navab M, Harkema J, Sioutas C, Lusis AJ, Nel AE (2008). Ambient particulate pollutants in the ultrafine range promote early atherosclerosis and systemic oxidative stress. Circ Res.

[9] Xia T, Kovochich M, Brant J, Hotze M, Sempf J, Oberley T, Sioutas C, Yeh JI, Wiesner MR, Nel AE (2006). Comparison of the abilities of ambient and manufactured nanoparticles to induce cellular toxicity according to an oxidative stress paradigm. Nano Lett.

[10] Nel AE, Madler L, Velegol D, Xia T, Hoek EM, Somasundaran P, Klaessig F, Castranova V, Thompson M (2009). Understanding biophysicochemical interactions at the nano-bio interface. Nat Mater.

[11] Suzuki Y, Tada-Oikawa S, Ichihara G, Yabata M, Izuoka K, Suzuki M, Sakai K, Ichihara S (2014). Zinc oxide nanoparticles induce migration and adhesion of monocytes to endothelial cells and accelerate foam cell formation. Toxicol Appl Pharmacol.

[12] Service RF (2003). American chemical society meeting. Nanomaterials show signs of toxicity. Science (New York, NY).

[13] Dillon AC (2010). Carbon nanotubes for photoconversion and electrical energy storage. Chem Rev.

[14] Vesterdal LK, Jantzen K, Sheykhzade M, Roursgaard M, Folkmann JK, Loft S, Møller P (2014). Pulmonary exposure to particles from diesel exhaust, urban dust or single-walled carbon nanotubes and oxidatively damaged DNA and vascular function in apoE(−/−) mice. Nanotoxicology.

[15] Guo YY, Zhang J, Zheng YF, Yang J, Zhu XQ (2011). Cytotoxic and genotoxic effects of multi-wall carbon nanotubes on human umbilical vein endothelial cells in vitro. Mutat Res.

[16] Bazile DV, Ropert C, Huve P, Verrecchia T, Marlard M, Frydman A, Veillard M, Spenlehauer G (1992). Body distribution of fully biodegradable [14C]-poly(lactic acid) nanoparticles coated with albumin after parenteral administration to rats. Biomaterials.

[17] Cagle DW, Kennel SJ, Mirzadeh S, Alford JM, Wilson LJ (1999). In vivo studies of fullerene-based materials using endohedral metallofullerene radiotracers. Proc Natl Acad Sci U S A.

[18] Asahara T, Masuda H, Takahashi T, Kalka C, Pastore C, Silver M, Kearne M, Magner M, Isner JM (1999). Bone marrow origin of endothelial progenitor cells responsible for postnatal vasculogenesis in physiological and pathological neovascularization. Circ Res.

[19] Du F, Zhou J, Gong R, Huang X, Pansuria M, Virtue A, Li X, Wang H, Yang XF (2012). Endothelial progenitor cells in atherosclerosis. Front Biosci (Landmark Ed).

[20] Walker VG, Li Z, Hulderman T, Schwegler-Berry D, Kashon ML, Simeonova PP (2009). Potential in vitro effects of carbon nanotubes on human aortic endothelial cells. Toxicol Appl Pharmacol.

[21] Orecna M, De Paoli SH, Janouskova O, Tegegn TZ, Filipova M, Bonevich JE, Holada K, Simak J (2014). Toxicity of carboxylated carbon nanotubes in endothelial cells is attenuated by stimulation of the autophagic flux with the release of nanomaterial in autophagic vesicles. Nanomedicine.

[22] Cao Y, Jacobsen NR, Danielsen PH, Lenz AG, Stoeger T, Loft S, Wallin H, Roursgaard M, Mikkelsen L, Møller P (2014). Vascular effects of multiwalled carbon nanotubes in dyslipidemic ApoE−/− mice and cultured endothelial cells. Toxicol Sci.

[23] Pacurari M, Qian Y, Fu W, Schwegler-Berry D, Ding M, Castranova V, Guo NL (2012). Cell permeability, migration, and reactive oxygen species induced by multiwalled carbon nanotubes in human microvascular endothelial cells. J Toxicol Environ Health A.

[24] Charo IF, Taubman MB (2004). Chemokines in the pathogenesis of vascular disease. Circ Res.

[25] Lu C, Shimaoka M, Salas A, Springer TA (2004). The binding sites for competitive antagonistic, allosteric antagonistic, and agonistic antibodies to the I domain of integrin LFA-1. J Immunol.

[26] Atarashi K, Hirata T, Matsumoto M, Kanemitsu N, Miyasaka M (2005). Rolling of Th1 cells via P-selectin glycoprotein ligand-1 stimulates LFA-1-mediated cell binding to ICAM-1. J Immunol.

[27] Xu YY, Yang J, Shen T, Zhou F, Xia Y, Fu JY, Meng J, Zhang J, Zheng YF, Yang J, Xu LH, Zhu XQ (2012). Intravenous administration of multi-walled carbon nanotubes affects the formation of atherosclerosis in Sprague–Dawley rats. J Occup Health.

[28] Li Z, Hulderman T, Salmen R, Chapman R, Leonard SS, Young SH, Shvedova A, Luster MI, Simeonova PP (2007). Cardiovascular effects of pulmonary exposure to single-wall carbon nanotubes. Environ Health Perspect.

[29] Han SG, Howatt D, Daugherty A, Gairola G (2015). Pulmonary and atherogenic effects of multi-walled carbon nanotubes (MWCNT) in apolipoprotein-E-deficient mice. J Toxicol Environ Health A.

[30] Poulsen SS, Jackson P, Kling K, Knudsen KB, Skaug V, Kyjovska ZO, Thomsen BL, Clausen PA, Atluri R, Berthing T, Bengtson S, Wolff H, Jensen KA, Wallin H, Vogel U (2016). Multi-walled carbon nanotube physicochemical properties predict pulmonary inflammation and genotoxicity. Nanotoxicology.

[31] Takahashi T, Kalka C, Masuda H, Chen D, Silver M, Kearney M, Magner M, Isner J, Asahara T (1999). Ischemia- and cytokine-induced mobilization of bone marrow-derived endothelial progenitor cells for neovascularization. Nat Med.

[32] Urbich C, Heeschen C, Aicher A, Dernbach E, Zeiher AM, Dimmeler S (2003). Relevance of monocytic features for neovascularization capacity of circulating endothelial progenitor cells. Circulation.

[33] Hill JM, Zalos G, Halcox JP, Schenke WH, Waclawiw MA, Quyyumi AA, Finkel T (2003). Circulating endothelial progenitor cells, vascular function, and cardiovascular risk. N Engl J Med.

[34] Schmidt-Lucke C, Rössig L, Fichtlscherer S, Vasa M, Britten M, Kämper U, Dimmeler S, Zeiher AM (2005). Reduced number of circulating endothelial progenitor cells predicts future cardiovascular events: proof of concept for the clinical importance of endogenous vascular repair. Circulation.

[35] O’Toole TE, Hellmann J, Wheat L, Haberzettl P, Lee J, Conklin DJ, Bhatnagar A, Pope CA (2010). Episodic exposure to fine particulate air pollution decreases circulating levels of endothelial progenitor cells. Circ Res.

[36] Haberzettl P, Lee J, Duggineni D, McCracken J, Bolanowski D, O’Toole TE, Bhatnagar A, Conklin DJ (2012). Exposure to ambient air fine particulate matter prevents VEGF-induced mobilization of endothelial progenitor cells from the bone marrow. Environ Health Perspect.

[37] Yang JX, Tang WL, Wang XX (2010). Superparamagnetic iron oxide nanoparticles may affect endothelial progenitor cell migration ability and adhesion capacity. Cytotherapy.

[38] Tada-Oikawa S, Ichihara G, Suzuki Y, Izuoka K, Wu W, Yamada Y, Mishima T, Ichihara S (2015). Zn(II) released from zinc oxide nano/micro particles suppresses vasculogenesis in human endothelial colony-forming cells. Toxicol Rep.

[39] Liberda EN, Cuevas AK, Gillespie PA, Grunig G, Qu Q, Chen LC (2010). Exposure to inhaled nickel nanoparticles causes a reduction in number and function of bone marrow endothelial progenitor cells. Inhal Toxicol.

[40] Patlolla AK, Patra PK, Flountan M, Tchounwou PB (2016). Cytogenetic evaluation of functionalized single-walled carbon nanotube in mice bone marrow cells. Environ Toxicol.

[41] Kreyling WG, Semmler M, Erbe F, Mayer P, Takenaka S, Schulz H, Oberdörster G, Ziesenis A (2002). Translocation of ultrafine insoluble iridium particles from lung epithelium to extrapulmonary organs is size dependent but very low. J Toxicol Environ Health A.

[42] Choi HS, Ashitate Y, Lee JH, Kim SH, Matsui A, Insin N, Bawendi MG, Semmler-Behnke M, Frangioni JV, Tsuda A (2010). Rapid translocation of nanoparticles from the lung airspaces to the body. Nat Biotechnol.

[43] Johnston HJ, Hutchison GR, Christensen FM, Peters S, Hankin S, Aschberger K, Stone V (2010). A critical review of the biological mechanisms underlying the in vivo and in vitro toxicity of carbon nanotubes: The contribution of physico-chemical characteristics. Nanotoxicology.

[44] Wu W, Ichihara G, Suzuki Y, Izuoka K, Oikawa-Tada S, Chang J, Sakai K, Miyazawa K, Porter D, Castranova V, Kawaguchi M, Ichihara S (2013). Dispersion method for safety research on manufactured nanomaterials. Ind Health.

[45] Murdock RC, Braydich-Stolle L, Schrand AM, Schlager JJ, Hussain SM (2008). Characterization of nanomaterial dispersion in solution prior to in vitro exposure using dynamic light scattering technique. Toxicol Sci.

[46] Inanaga K, Ichiki T, Miyazaki R, Takeda K, Hashimoto T, Matsuura H, Sunagawa K (2010). Acetylcholinesterase inhibitors attenuate atherogenesis in apolipoprotein E-knockout mice. Atherosclerosis.

[47] Endtmann C, Ebrahimian T, Czech T, Arfa O, Laufs U, Fritz M, Wassmann K, Werner N, Petoumenos V, Nickenig G, Wassmann S (2011). Angiotensin II impairs endothelial progenitor cell number and function in vitro and in vivo : implications for vascular regeneration. Hypertension.

[48] Chen YH, Lin SJ, Lin FY, Wu TC, Tsao CR, Huang PH, Liu PL, Chen YL, Chen JW (2007). High glucose impairs early and late endothelial progenitor cells by modifying nitric oxide-related but not oxidative stress-mediated mechanisms. Diabetes.

