# Supplementary material for: Single- and double-walled carbon nanotubes enhance atherosclerogenesis by promoting monocyte adhesion to endothelial cells and endothelial progenitor cell dysfunction
Source: Part Fibre Toxicol. 2016 Oct 13;13:54. doi: 10.1186/s12989-016-0166-0 (PMC5064793; doi:10.1186/s12989-016-0166-0)
Supplement: Additional file 1: Figure S1. — Effects of SWCNTs and DWCNTs on cell viability. (PPTX 70 kb) [file 12989_2016_166_MOESM1_ESM.pptx]

## Slide 1
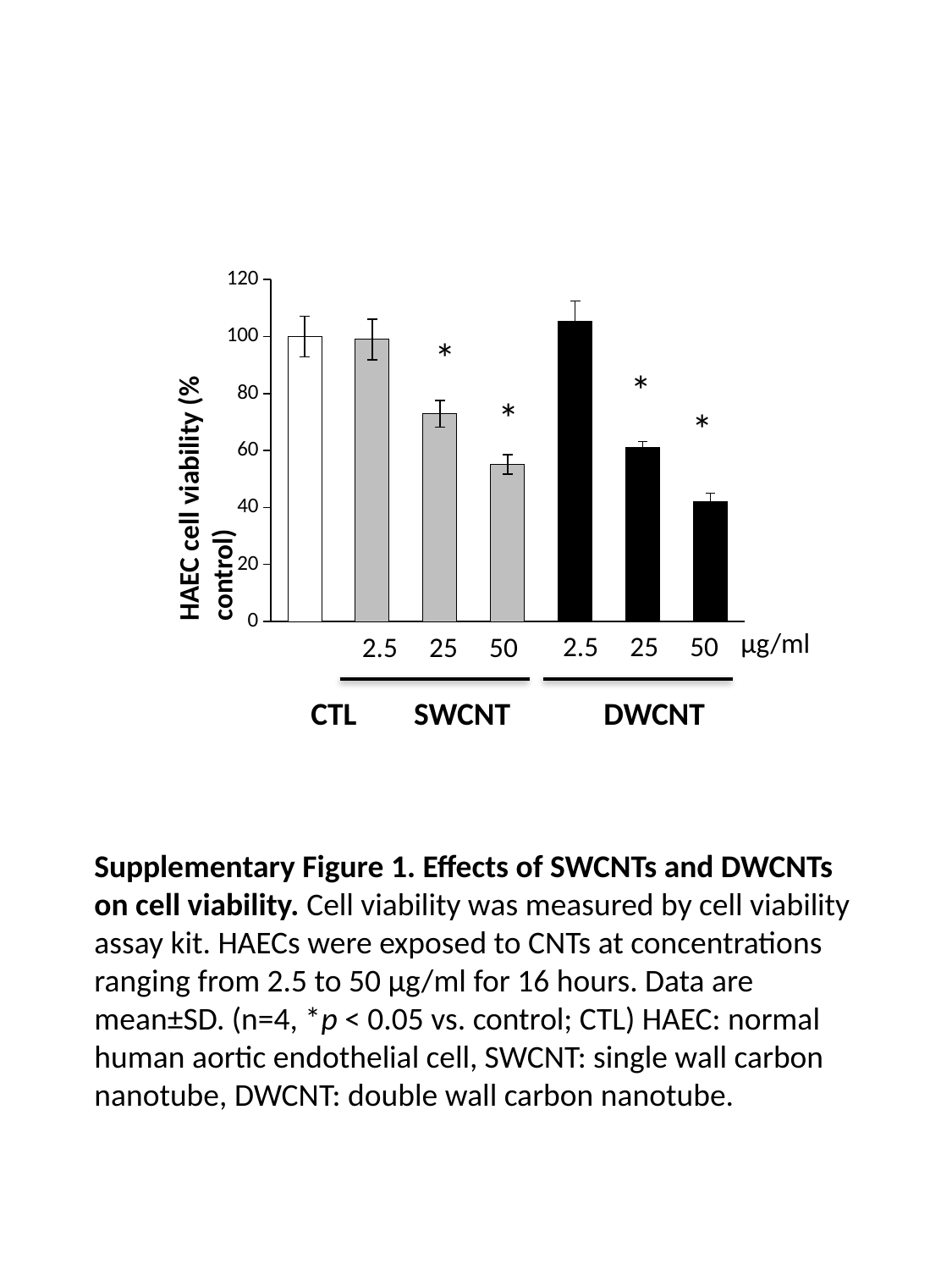

### Chart
| Category | |
|---|---|
| CTL | 100.0 |
| S2.5 | 98.95833333333334 |
| S25 | 72.91666666666666 |
| S50 | 55.104166666666664 |
| D2.5 | 105.20833333333333 |
| D25 | 61.04166666666666 |
| D50 | 42.08333333333332 |HAEC cell viability (% control)
*
*
*
*
μg/ml
2.5 25 50
2.5 25 50
CTL SWCNT DWCNT
Supplementary Figure 1. Effects of SWCNTs and DWCNTs on cell viability. Cell viability was measured by cell viability assay kit. HAECs were exposed to CNTs at concentrations ranging from 2.5 to 50 μg/ml for 16 hours. Data are mean±SD. (n=4, *p < 0.05 vs. control; CTL) HAEC: normal human aortic endothelial cell, SWCNT: single wall carbon nanotube, DWCNT: double wall carbon nanotube.
